# Neuromyths in Education: Prevalence among Spanish Teachers and an Exploration of Cross-Cultural Variation

**DOI:** 10.3389/fnhum.2016.00496

**Published:** 2016-10-13

**Authors:** Marta Ferrero, Pablo Garaizar, Miguel A. Vadillo

**Affiliations:** ^1^Experimental Psychology, Division of Psychology and Language Sciences, University College LondonLondon, UK; ^2^Colegio Berrio-OtxoaBilbao, Spain; ^3^Faculty of Engineering, University of DeustoBilbao, Spain; ^4^Primary Care and Public Health Sciences, King’s College LondonLondon, UK

**Keywords:** neuromyths, teachers, education, neuroscience, meta-analysis

## Abstract

Enthusiasm for research on the brain and its application in education is growing among teachers. However, a lack of sufficient knowledge, poor communication between educators and scientists, and the effective marketing of dubious educational products has led to the proliferation of numerous ‘neuromyths.’ As a first step toward designing effective interventions to correct these misconceptions, previous studies have explored the prevalence of neuromyths in different countries. In the present study we extend this applied research by gathering data from a new sample of Spanish teachers and by meta-analyzing all the evidence available so far. Our results show that some of the most popular neuromyths identified in previous studies are also endorsed by Spanish teachers. The meta-analytic synthesis of these data and previous research confirms that the popularity of some neuromyths is remarkably consistent across countries, although we also note peculiarities and exceptions with important implications for the development of effective interventions. In light of the increasing popularity of pseudoscientific practices in schools worldwide, we suggest a set of interventions to address misconceptions about the brain and education.

## Introduction

Over the last few decades, the scientific community has shown an increasing interest in building bridges between brain science and education. This interest has promoted the creation of research teams, specialized journals, and meetings aimed at connecting neuroscience and educational practice (Edelenbosch et al., 2015). The enthusiasm for neuroscience has spread amongst educational practitioners (Pickering and Howard-Jones, 2007; Ansari et al., 2012), who see it as great opportunity to improve or enrich their way of teaching (Simmonds, 2014). However, within the scientific community there is a clear consensus that calls to increase the use of neuroscientific research in classrooms are premature (Goswami, 2004, 2006; Blakemore and Frith, 2005; Lindell and Kidd, 2011). By contrast, the prevailing view among teachers is that neuroscience is ready to directly inform curricular decisions (Goswami, 2004, 2006).

The diverging views on the potential contributions of neuroscience to education among scientists and educational practitioners are due to several reasons (Samuels, 2009). For instance, the differences in the language and terminology used by researchers and teachers are substantial (Goswami, 2006; Varma et al., 2008; Christodoulou and Gaab, 2009; Howard-Jones, 2014). In addition, collaboration between scientists and educators is still rare (Goswami, 2006; Pickering and Howard-Jones, 2007; Fisher et al., 2010; Ansari et al., 2011; Howard-Jones, 2014; Edelenbosch et al., 2015; Schwartz, 2015). Moreover, teachers lack scientific knowledge and skills to critically evaluate neuroscientific claims and distinguish evidence-based from non-evidence-based practices (Lindell and Kidd, 2011; Lilienfeld et al., 2012; Busso and Pollack, 2014). This gap between researchers and practitioners has caused the misinterpretation and oversimplification of scientific research and facilitated the rapid proliferation of several misconceptions about the mind and the brain, known as neuromyths (Organization for Economic Co-operation and Development [OECD], 2002, 2007; Goswami, 2006; Waterhouse, 2006; Geake, 2008; Kalbfleisch and Gillmarten, 2013; Howard-Jones, 2014). Like other misconceptions (Lilienfeld et al., 2012), neuromyths may contain a kernel of truth, but are oversimplified or misunderstood. In addition, the studies that have addressed other socially relevant misconceptions, like anti-vaccination myths, have found that correcting them is a daunting challenge because misinformation is highly resistant to change (Nyhan et al., 2014; Nyhan and Reifler, 2015).

Several factors have contributed to the spread of neuromyths across schools. On the one hand, the inclusion of neuroscientific content encourages lay people to believe that psychological explanations are more scientifically sound (Racine et al., 2005; Weisberg et al., 2008; Lindell and Kidd, 2013). On the other hand, the diffusion of publications, conferences, workshops, or educational materials prepared by non-specialists has facilitated the proliferation of neuroscientific content of questionable validity throughout the educational community (Goswami, 2006; Busso and Pollack, 2014; Simmonds, 2014). In a similar way, there has been an exponential grown of “brain-based” commercial programs that have popularized pseudoscientific practices in schools (Goswami, 2006; Sylvan and Christodoulou, 2010). For example, the program Brain Gym^®^ is offered in more than 80 countries (Hyatt, 2007) and is employed by more than 900 schools only in the United Kingdom (UK; Goldacre, 2006). Similarly, the neuro-educational program “Brain Training” represents a $300 million-a-year industry in the USA alone (Hurley, 2012). These practices are not completely invalid but the statements linked to them considerably exceed the available evidence (Lilienfeld et al., 2012). The propagation of brain-based interventions with dubious scientific basis involves not only a substantial economic cost, but also an opportunity cost; that is, parents and children risk wasting money and time in a useless treatment when they could invest those resources on an effective solution (Busso and Pollack, 2014). Many of these practices are unlikely to produce any benefit and can even harm schoolchildren (Lilienfeld, 2007; Pasquinelli, 2012).

Concerned about the proliferation of neuromyths inside the educational community, Howard-Jones et al. (2009) surveyed trainee teachers with a questionnaire containing assertions about the brain (Herculano-Houzel, 2002) and several common neuromyths (Pickering and Howard-Jones, 2007). The results showed that more than half of the sample approved a substantial number of myths about the brain (Howard-Jones et al., 2009). Using a similar survey, Dekker et al. (2012) found a high prevalence of neuromyths among primary and secondary school teachers in the UK and the Netherlands, although there was some variation in which neuromyths were most prevalent. The surveys developed by Howard-Jones et al. (2009) and Dekker et al. (2012) have also been applied to educators in Greece (Deligiannidi and Howard-Jones, 2015), China (Pei et al., 2015), Turkey (Karakus et al., 2015), and Latin America (Gleichgerrcht et al., 2015), and to trainee teachers in Spain (Fuentes and Risso, 2015). In addition, a similar questionnaire has been applied in Portugal (Rato et al., 2013). As in the previous research, these studies confirmed a high popularity of neuromyths among the teachers in all of these countries.

In Spain, the prevalence of courses, conferences, and educational programs related to neuroscience has increased in recent years. For instance, a new project called HERAT (Spanish acronym for hydration, balance, breathing, attention, and touch), with similar characteristics to Brain Gym^®^, has been set up in 30 schools since it was launched last academic year. According to its authors, it consists on a set of exercises aimed at “activating the brain, promoting neurological reorganization and facilitating learning in the whole brain” (Proyecto NeuroEducacióN en Educación Infantil, n.d.). This acceptance of pseudo-neuroscientific content suggests that Spanish teachers are just as fascinated with neuroscience as educators from other countries (Pickering and Howard-Jones, 2007). As mentioned above, there is evidence that novice trainee teachers in some regions of Spain believe in a considerable number of neuromyths (Fuentes and Risso, 2015). However, currently there is no evidence on the prevalence of misconceptions about the brain among in-service teachers in this country.

The purpose of the present study was to determine the prevalence of neuromyths among teachers from all levels of school education. As in previous studies, we were also interested in investigating which myths are more or less popular in this population. A better knowledge on the subject would be helpful to design more effective interventions to address neuromyths among Spanish teachers. In addition, we were interested in determining which factors predict belief in neuromyths. Specifically, we aimed to determine whether reading popular or scientific neuroscience literature prevents teachers from believing in neuromyths or, by contrast, if greater knowledge about the brain is associated with higher acceptance of misconceptions, as has been shown in previous studies (Dekker et al., 2012; Gleichgerrcht et al., 2015). To provide valuable information for future prevention programs, we sought to explore the origins of teachers’ incorrect ideas (e.g., books, schools, web sites). Additionally, this study examined the connection between a range of teacher characteristics (e.g., age, sex, years of experience) with general knowledge and belief in neuromyths.

A second goal of the present study was to combine data collected in our Spanish sample and the evidence gathered in previous studies, to explore similarities and differences in the popularity of neuromyths across countries. As explained above, some of the previous studies on this topic have addressed the issue of cross-cultural variability in neuromyths by collecting data simultaneously from different countries (e.g., Dekker et al., 2012; Gleichgerrcht et al., 2015). However, the analysis of within-study variability neglects the rich information gathered so far across different studies. In the present article, we synthesized the evidence from all the previous studies that explored the prevalence of neuromyths among teachers using the questionnaires originally devised by Howard-Jones et al. (2009) and Dekker et al. (2012). Our goal was to quantify cross-national variability in the popularity of each neuromyth using meta-analytic methods to obtain a clearer view of the particular idiosyncrasies of each country.

## Materials and Methods

### Participants

The sample included 284 teachers from 15 independent Spanish regions (out of a total of 19). Eighty (28.07%) participants were males and 204 (71.57%) females. These percentages are proportional to the distribution of males and females in the total population of educators in Spain (Instituto Nacional de Estadística, 2015). The mean age of participants was 42.1 years (*SD* = 9.28). Participants were kindergarten teachers (22.8%), primary school teachers (32.9%), secondary school teachers (33.6%), vocational education teachers (3.5%), and teachers who worked in more than one level of education (6.3%). The sample was recruited from public (27.7%), private (6.3%), and state schools (64.9%). The average teaching experience of the participants was 16.9 years (*SD* = 9.69). Except for gender, age, and years of experience, these demographic and professional data were requested only for descriptive purposes and were not explored any further in subsequent analyses.

### Procedure

Teachers were contacted by email invitation to pseudo-randomly selected databases of schools available on the Internet, by personal invitation, or by two social networking sites. In the first case, after accepting the invitation, schools were asked to forward an email with information about the study to all their staff teachers. In the remaining two cases, information about the study along with an invitation to participate was sent by email, in the case of the direct invitation, or was published simultaneously on Twitter and Facebook, in the case of the social networking sites. In all the modalities, teachers who were interested in participating followed a link to the on-line survey. The research project was presented to the participants as a study about the role of neuroscience in education. Average completion time for each survey was approximately 15 min.

### Materials

The survey consisted of two parts. In the first part, participants gave their informed consent and provided background information about their age, sex, professional qualification (e.g., degree, master degree, PhD), years of teaching, level at which they teach (e.g., kindergarten, elementary school, secondary school), position within the school (e.g., teacher, coordinator, headmaster), type of school they attended (e.g., public school, private school) and the region where their school was located. Teachers indicated whether they were interested in neuroscience applied to education and whether they thought this knowledge was important for their job. In addition, they were asked whether they had received in-service training in educational neuroscience (e.g., learning styles, multiple intelligences, left/right brain learners) and, if so, how they got to known of its existence and the institution that had organized it. Additionally, they were asked whether any of the so-called ‘brain-based’ programs was being implemented in their schools. Furthermore, they indicated whether they read magazines about general or educational science and/or peer-review journal articles. To avoid misunderstandings, well-known examples of each type of publication were given. Finally, teachers were asked to provide information about any book, blog, or website about neuroscience that they consulted regularly.

In the second part, participants completed the survey developed by Dekker et al. (2012). This survey included a set of educational neuromyths as defined by OECD (2002) and Howard-Jones et al. (2009), together with additional, general statements about the brain. The rationale for Dekker et al. (2012) to consider ‘neuromyths’ and ‘general knowledge questions’ separately is that the ‘neuromyth’ items specifically address a number of beliefs that had been identified as such by the OECD, while the ‘general knowledge questions’ were not mentioned in the original OECD report. For the sake of consistency with previous studies, in the present study we also analyzed neuromyths and general knowledge questions separately. The questionnaire consisted of 32 statements in total: 12 about neuromyths and 19 general assertions about the brain^1^ (see **Tables 1** and **2**), presented in random order. Participants were instructed to respond by marking one of three options: *correct. incorrect*, or *do not know*. All the neuromyths were false statements about the brain, while the general knowledge questions comprised true and false statements (see **Table 2**). The original survey was initially translated into Spanish by one researcher (MF). To guarantee the fidelity of the translation, the resultant version was back-translated into English by a second researcher (MAV) and both English versions were compared by a third person.

**Table 1:** Percentage of correct and incorrect responses for each neuromyth.

	Incorrect (%)	Correct (%)	Do not know (%)
Environments that are rich in stimulus improve the brains of pre-school children.	94	2.8	3.1
Individuals learn better when they receive information in their preferred learning style (e.g., auditory, visual, and kinesthetic).	91.1	4.9	3.8
Exercises that rehearse coordination of motor-perception skills can improve literacy skills.	82	3.5	14.4
Short bouts of coordination exercises can improve integration of left and right hemispheric brain function.	77.1	1.7	21.1
Differences in hemispheric dominance (left brain, right brain) can help explain individual differences among learners.	67.2	10.2	22.5
It has been scientifically proven that fatty acid supplements (omega-3 and omega-6) have a positive effect on academic achievement.	45	10.5	44.3
We only use 10% of our brain.	44	32.7	23.2
Children are less attentive after consuming sugary drinks and/or snacks.	33.8	27.8	38.3
There are critical periods in childhood after which certain things can no longer be learned.	29.9	56.6	13.3
Children must acquire their native language before a second language is learned. If they do not do so neither language will be fully acquired.	10.9	80.2	8.8
If students do not drink sufficient amounts of water (=6–8 glasses a day) their brains shrink.	7.7	64.7	27.4
Learning problems associated with developmental differences in brain function cannot be remediated by education.	7	78.5	14.4




**Table 2:** Percentage of correct and incorrect responses for each general assertion about the brain.

	Incorrect (%)	Correct (%)	Do not know (%)
The left and right hemispheres of the brain always work together. (T)	61.9	21.4	16.5
Boys have bigger brains than girls. (T)	57.3	8	34.5
When a brain region is damaged other parts of the brain can take up its function. (T)	28.5	50	21.4
Regular drinking of caffeinated drinks reduces alertness. (T)	24.2	36.9	38.7
Circadian rhythms (“body clock”) shift during adolescence, causing pupils to be more tired during the first lessons of the school day. (T)	22.2	36.2	41.5
The brains of boys and girls develop at the same rate. (F)	20.7	51.7	27.4
Vigorous exercise can improve mental function. (T)	15.1	55.6	29.2
Information is stored in the brain in a network of cells distributed throughout the brain. (T)	14	41.9	44
Extended rehearsal of some mental processes can change the shape and structure of some parts of the brain. (T)	13.3	55.9	30.6
Normal development of the human brain involves the birth and death of brain cells. (T)	13.3	63.7	22.8
Academic achievement can be affected by skipping breakfast. (T)	11.6	79.2	9.1
Learning occurs through modification of the brains’ neural connections. (T)	6.3	67.2	26.4
Brain development has finished by the time children reach secondary school. (F)	5.6	79.9	14.4
There are sensitive periods in childhood when it is easier to learn things. (T)	4.9	86.2	8.8
We use our brains 24 h a day. (T)	4.5	93.6	1.7
Production of new connections in the brain can continue into old age. (T)	3.8	78.5	17.6
Individual learners show preferences for the mode in which they receive information (e.g., visual, auditory, kinesthetic). (T)	2.1	93.6	4.2
Mental capacity is hereditary and cannot be changed by the environment or experience. (F)	1	96.4	2.4
When we sleep, the brain shuts down. (F)	0	98.23	1.7


*(T), True; (F), False.*

### Data Analysis

The α value for all statistical tests was set to 0.05. To examine which factors predicted belief in neuromyths, we conducted a multiple regression analysis with the number of myths as the dependent variable. The predictors were sex, age, years of experience, in-service training in educational neuroscience, reading popular magazines of science or education, reading scientific journals, consulting of blogs or web sites, and number of correct answers on general assertions about the brain. To examine the predictors of general knowledge about the brain, a second analysis was conducted with the percentage of correct answers to the general assertions as the dependent variable. In this case, the predictors were sex, age, years of experience, in-service training, reading popular magazines of science or education, reading scientific journals, and consulting of blogs and web sites.

As a means to visualize similarities and differences across countries, we meta-analyzed the proportion of incorrect responses to each neuromyth using the data gathered in the present study and the results of previous studies exploring the prevalence of neuromyths in teachers of different countries (Dekker et al., 2012; Deligiannidi and Howard-Jones, 2015; Gleichgerrcht et al., 2015; Karakus et al., 2015; Pei et al., 2015). Specifically, we conducted a separate meta-analysis for each neuromyth, using as the dependent variable the proportion of people endorsing the myths in each country (see **Table S2** in the Supplementary Material). Given that proportions do not follow a normal distribution, we conducted the (random effects) meta-analyses on double arcsine transformed proportions (Freeman and Tukey, 1950) and then we back-transformed the meta-analytic estimates and confidence intervals to proportions (Miller, 1978). The meta-analysis was conducted with the metafor R package (Viechtbauer, 2010). The amount of cross-cultural variability was quantified with the popular *I*^2^ index, which measures the proportion of variance that must be attributed to systematic differences across studies rather than to chance (Higgins et al., 2003). Systematic reviews typically assess the quality of each study entered into a meta-analysis taking into account different methodological features like risk of bias or sample size (Jüni et al., 1999; Higgins et al., 2011). In the present case, all the studies rely on the same procedure and materials and, consequently, share the same methodological shortcomings. The only important difference between them is that they are based on very different sample sizes and sampling methods. The interested reader can find detailed information about these features in **Table S1** of the Supplementary Material.

## Results

### Professional Profile and Interest in Neuroscience

Overall, 98.5% teachers were interested in the brain and its role on learning, and 95.4% considered scientific knowledge about the brain very important for their teaching practice. A total of 29.5% of teachers stated they read popular magazines about science, 42.6% reported they read popular magazines about education, and 7% stated they read primary scientific journals. Additionally, 96.1% had access to information about education and the brain using other sources such as web pages and blogs (51.4%), books (27.8%), or in-training courses (16.9%). Among the latter, 52% knew about the existence of the courses from their own schools, 25% from the internet, and the remaining 22.9% from different sources such as the press, university, or friends. The most prevalent in-service training course topic was the multiple intelligences theory, which is not based on solid scientific evidence (Waterhouse, 2006). Of all teachers, 71.12% stated having encountered educational approaches that claimed to be brain-based in their respective schools.

### Prevalence of Neuromyths

**Table 1** summarizes the proportion of correct and incorrect responses for each neuromyth. For the overall sample, teachers failed to recognize 49.1% (*SD* = 17%) of neuromyths on average. In addition, 19.6% neuromyths (*SD* = 16.8%) were labeled as *do not know*. The most prevalent neuromyths were (1) “environments that are rich in stimulus improve the brains of pre-school children,” believed by 94% teachers; (2) “individuals learn better when they receive information in their preferred learning style,” believed by 91.1% teachers; and (3) “exercises that rehearse coordination of motor-perception skills can improve literacy skills,” believed by 82% teachers. In contrast, the most successfully identified neuromyths were (1) “children must acquire their native language before a second language is learned,” marked as false by 80.2% teachers; (2) “learning problems associated with developmental differences in brain function cannot be remediated by education,” marked as false by 78.5% teachers; and (3) “if students do not drink sufficient amounts of water their brains shrink,” marked as false by 64.7% teachers. Due to the important role bilingualism plays in some regions of Spain, the responses to the myth about the importance of acquiring a native language before learning a second language were analyzed separately for regions with several official languages. The results showed that among teachers who belonged to a region with just one official language, 52.3% believed the myth, while among the teachers who belonged to a region with two official languages, 76.9% believed the myth.

The multiple regression analysis revealed that women were more likely to believe in neuromyths that men (unstandardised *B* = 0.99). In addition, belief in neuromyths was predicted by general knowledge of the brain: Teachers who responded correctly to general knowledge questions about the brain were also more likely to believe in neuromyths (*B* = 0.31). Importantly, while having read scientific journals reduced belief in neuromyths (*B* = -0.92), having read educational magazines actually increased this belief (*B* = 0.52). The remaining factors did not predict belief in neuromyths (see **Table 3**).

**Table 3:** Predictors of neuromyths.

	*B* (*SE*)	*t*	*p*	95% CI for *B*	
				
				Lower	Upper
Age	0.000 (0.028)	0.016	0.987	-0.054	0.055
Gender	0.994 (0.240)	4.136	0.000^∗∗^	0.521	1.467
Experience	-0.030 (0.026)	-1.128	0.261	-0.082	0.022
In-service training	0.161 (0.322)	0.500	0.618	-0.473	0.795
Read science magazines	-0.169 (0.249)	-0.678	0.498	-0.658	0.321
Read education magazines	0.522 (0.231)	2.263	0.024^∗^	0.068	0.976
Read scientific journals	-0.922 (0.449)	-2.052	0.041^∗^	-1.807	-0.037
Read books	-0.105 (0.268)	-0.394	0.694	-0.632	0.421
Consult webs and blogs	-0.307 (0.222)	-1.383	0.168	-0.745	0.130
Knowledge (#correct)	0.315 (0.038)	8.381	0.000^∗∗^	0.241	0.389


*^∗^*p* < 0.05, ^∗∗^*p* < 0.01.*

### Knowledge about the Brain

Teachers responded correctly to 62.29% (*SD* = 16.04%) of the general statements about the brain and labeled 20.72% (*SD* = 16.8%) as *do not know* (see **Table 2**). Knowledge about the brain was predicted by reading primary scientific journals (*B* = 1.56), by in-service training (*B* = 1.38), and by reading books about neuroscience (*B* = 1.06). The remaining factors did not predict general knowledge of the brain (see **Table 4**).

**Table 4:** Predictors of general knowledge.

	*B* (*SE*)	*t*	*p*	95% CI for *B*	
				
				Lower	Upper
Age	0.038 (0.044)	0.848	0.397	-0.050	0.125
Gender	-0.687 (0.387)	-1.776	0.077	-1.448	0.075
Experience	-0.045 (0.043)	-1.067	0.289	-0.129	0.039
In-service training	1.383 (0.515)	2.687	0.008^∗∗^	0.370	2.396
Read science magazines	0.131 (0.402)	0.325	0.745	-0.661	0.922
Read education magazines	0.544 (0.372)	1.464	0.144	-0.188	1.277
Read scientific journals	1.566 (0.721)	2.172	0.031^∗^	0.146	2.985
Read books	1.066 (0.428)	2.491	0.013^∗^	0.224	1.909
Consult webs and blogs	-0.263 (0.359)	-0.731	0.466	-0.970	0.445


*^∗^*p* < 0.05, ^∗∗^*p* < 0.01.*

### Similarities and Differences across Countries

**FIGURE 1:**
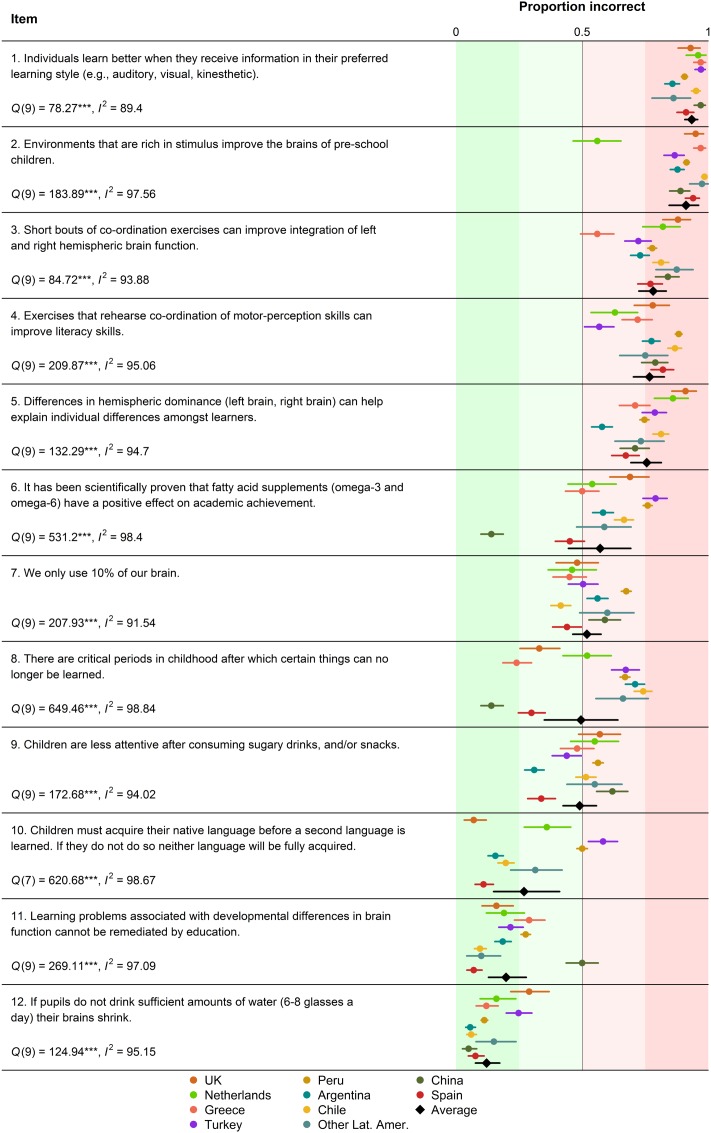
**Proportion of participants believing in each neuromyth across different countries.** The error bars denote 95% confidence intervals. The black diamonds represent the meta-analytic random-effects estimates for each item. See the main text for more details.

**Figure 1** shows the prevalence of each neuromyth in the different countries where studies like the present one have been conducted so far. The forest plots reveal some interesting consistencies across countries. For instance, the idea that pupils learn better when taught in their preferred learning style and the idea that rich environments improve the brains of pre-school children are extraordinarily popular in most countries. The prevalence of the former ranges from 85.8 to 97.1% across countries and the prevalence of the latter ranges from 86.7 to 98.5%, with the only exception of the Netherlands, which shows a lower prevalence.

Beyond these similarities, the *I*^2^ indexes confirm that there is a very large amount of cross-cultural variation. Even for the neuromyths with more consistent responses, at least 89.40% of the variance must be attributed to systematic differences across countries. For all items, the results of the *Q*-test show that the level of statistical heterogeneity is significantly larger than what would be expected by mere chance, confirming the presence of cross-cultural differences. To mention some noteworthy examples, according to the values of *I*^2^, the idea that there are critical periods of learning after which some types of learning become impossible (*I*^2^ = 98.84%) and the belief that it has been scientifically proven that fatty acid supplements can improve academic performance (*I*^2^ = 98.80%) are the items with the largest level of cross-cultural variation. The forest plots in **Figure 1** show that these myths are very popular in some countries (e.g., Turkey and Peru), but not in others (e.g., China).

## Discussion

Over the last years, cognitive neuroscience has gradually taken on the challenge of understanding the neural mechanisms that enable human learning (Meltzoff et al., 2009). However, the translation of neuroscience research to the education community has not been straightforward (Bruer, 1997; Blakemore and Frith, 2005) and misconceptions about neuroscientific claims are widespread amongst educational practitioners (Goswami, 2006). The present study examined the prevalence of these neuromyths among teachers of different regions in Spain as well as their general knowledge about the brain. In addition, we investigated if these outcomes were associated with factors such as demographic characteristics of teachers, or access to a diverse range of neuroscientific materials.

The results obtained in this study showed that Spanish teachers believed a considerable number of the neuromyths. Specifically, from a total of 12 neuromyths presented, five were believed by more than 50% of the educators. This result is very similar to the patterns observed in British and Dutch teachers and only slightly better than the results obtained in Latin America and Turkey. Conversely, the mean score on general knowledge questions about the brain was almost 60% amongst Spanish educators. This result is worse than the one obtained in the UK, Netherlands, and Latin America and could be due to the quality and quantity of the educational materials available in Spain. In this regard, as noted by Gleichgerrcht et al. (2015), the reduced access to material written in Spanish could play an important role.

As in previous studies (Dekker et al., 2012; Gleichgerrcht et al., 2015), teacher characteristics (e.g., age, years of teaching, in-service training) did not predict belief in neuromyths or general knowledge about the brain, with the exception of gender. On average, women believed more neuromyths than men. As previously reported by Dekker et al. (2012) and Gleichgerrcht et al. (2015), knowledge about the brain did not protect teachers from believing in neuromyths. On the contrary, educators who seemed to know more about the brain committed more errors in identifying neuromyths. This phenomenon may be explained by an acquiescence bias; that is, teachers who responded affirmatively to a greater number of general assertions about the brain also gave more affirmative answers to neuromyths. An alternative explanation, suggested by Dekker et al. (2012), is that teachers have difficulty discriminating correct and incorrect information about the brain to which they are exposed in their profession. In relation to this, we found that while having read scientific journals reduced belief in neuromyths, having read educational magazines increased this belief. Taken together, these results underline the relevance that quality of information has in teachers’ beliefs about the brain.

Along with the sources of information explored by the preceding studies, we were interested in analyzing other means Spanish educators employed to learn about the brain, such as books or web sites. Overall, the favorite sources of information reported by teachers contrasted with the ones that predicted knowledge about the brain. In other words, the resources that seem to promote general knowledge are the least popular among the teachers tested in the present study. Contrary to the results obtained by Gleichgerrcht et al. (2015) in Latin America, only a small percentage of teachers in the present study reported reading primary scientific journals. This difference can be explained by the manner in which the different kinds of publications were presented to our sample. Specifically, unlike in preceding studies, we accompanied each publication type with a couple of well-known examples in order to avoid misunderstandings. In the absence of these clarifications, it is possible that many teachers tested in previous studies reported reading scientific journals when they actually meant that they read popular science magazines. Furthermore, it is worth mentioning that more than half of the teachers who had taken a course about the brain and learning had done so through their own schools, which highlights the key role schools can play in the proliferation of neuromyths.

The eager interest in neuroscientific claims and their potential applications to education, together with the high prevalence of neuromyths amongst Spanish teachers, echoes the findings obtained in previous studies conducted in Asia, Europe, and Latin America (Dekker et al., 2012; Deligiannidi and Howard-Jones, 2015; Gleichgerrcht et al., 2015; Karakus et al., 2015; Pei et al., 2015). Nevertheless, the meta-analysis conducted in this study indicates that some neuromyths are markedly more widespread across countries than others (e.g., the need to adapt teaching to learning styles and the importance of environments rich in stimuli on the brain of pre-schoolers). The study conducted in Spain supports and extends these results, since these two myths are also the most popular among Spanish teachers (see **Supplementary Table S2**). It should be stressed that both myths are present in several commercial educational packages, which may have contributed to their dissemination. For instance, in the case of learning styles, there is a huge industry devoted to publishing measurement instruments and guidebooks, and to organizing workshops and conferences targeted mainly at teachers (Pashler et al., 2008; Kirschner and van Merriënboer, 2013). Similarly, the myth about the importance of rich environments has been disseminated by several books written by Glenn Doman, who has promoted his method for making babies more intelligent around the world (Edkin, 1987). Conversely, some neuromyths exhibit low prevalence across countries. This is the case for the misconception related to the importance of students drinking sufficient amounts of water to prevent their brains shrinking. This erroneous idea has been championed by Brain Gym^®^, a program which has found widespread acceptance only in some countries, such as the UK (Hyatt, 2007). Similarly, few teachers endorse the idea that learning problems associated with differences in brain function cannot be ameliorated by education or that children must acquire their native language before a second language is learned.

In light of the present study and the preceding ones, the prevalence of neuromyths among educators is not an isolated phenomenon but, on the contrary, affects many different countries around the world. Given the gap that exists between scientists and practitioners, many experts agree that it is essential to establish interdisciplinary collaboration between neuroscientists and educators to inform each other and to create useful connections in both fields (Ansari et al., 2011; Howard-Jones, 2014). In this regard, some organizations have already embarked on fostering collaboration between researchers and practitioners and promoting a better understanding of brain function in relation to education, such as the British and American Educational Research Associations (BERA, AERA), the Strategic Education Research Partnership (SERP), or the Centre for Neuroscience in Education. Additionally, some researchers have implemented a comprehensive set of actions, such as the organization of seminar series between scientists and educators (Pickering and Howard-Jones, 2007), the creation of teacher learning communities supported by education institutes and researchers (Hille, 2011), or the opening of research labs for teachers and student teachers to foster dialog between the different agents involved (Coch et al., 2009). Furthermore, some researchers have founded organizations like the International Brain, Mind and Education Society, which has promoted, among others, the development of master programs and the creation of a new journal embedded in this emerging field (Schwartz, 2015). In addition, some researchers have suggested the possibility of creating research schools as an infrastructure to support the mutual collaboration of scientists and practitioners (Hinton and Fisher, 2008) or the promotion of a new generation of researchers specialized in both scientific and educational methods (Goswami, 2004; Fisher et al., 2010). Finally, as a result of this emerging interest in enhancing links between research and educational practice, some experts have started elaborating papers to properly inform laypeople about some of the main findings of neuroscience applied to education (Kalbfleisch and Gillmarten, 2013; Gearin and Fien, 2016).

In Spain, several researchers have expressed concerns about neuromyths in educational contexts (Marina, 2012; Forés et al., 2015; Fuentes and Risso, 2015). However, to date there is a dearth of initiatives to prevent their proliferation. Now that a draft guide on the next educational reform is being prepared (Ministry of Education, Culture and Sport in Spain, 2015), it would be advisable to include the most prevalent myths about brain and education in both initial teacher training and ongoing professional development programs of Spanish educators. Based on the results of the present study, the list should specially include the myths related to the need to adapt teaching to learning styles, the importance of environments rich in stimuli on the brain of preschoolers, and the effectiveness of exercises that rehearse co-ordination of motor-perception skills on the improvement of literacy skills. Similarly, it would be convenient to add introductory content about neuroscience and research methodologies in these courses (Goswami, 2004; Ansari et al., 2011; Lilienfeld et al., 2012). These actions would empower Spanish teachers to think more critically about brain-based claims and to become more critical and thoughtful consumers of neuroscientific evidence (Lindell and Kidd, 2011; Lilienfeld et al., 2012). In addition, it would be necessary to address the lack of rigorous and digestible neuroscience contents developed for teachers, especially in the Spanish language (Gleichgerrcht et al., 2015). This could be achieved through the recruitment of research communicators who can interpret and inform teachers about the progress of neuroscience and, at the same time, provide feedback to researchers about questions, criticisms and proposals raised by educators (Goswami, 2006; Fisher et al., 2010). Finally, it might also be advisable that Spanish educational authorities work closely with neuroscience experts and practitioners (Schwartz, 2015) to ensure that brain-based training courses and programs offered in schools are based on solid scientific evidence about the brain and not on misunderstandings or oversimplifications of original research.

To this day, neuroscientific findings are valuable for describing the mechanisms of learning. However, they cannot yet inform educational practice directly (Goswami, 2004, 2006; Blakemore and Frith, 2005; Lindell and Kidd, 2011; Thomas, 2013). While some researchers keep a cautious but optimistic vision about the future of educational neuroscience (Ansari and Coch, 2006; Goswami, 2006; Varma et al., 2008; Thomas, 2013), others are more skeptical about the potential of neuroscience to improve teaching in the future (Bruer, 1997; Bowers, 2016). Only time will tell us which is the future of this emerging discipline. Meanwhile, it is advisable to be vigilant in the face of the appearance of new myths in the classrooms.

## Author Contributions

All authors developed the study concept. MF developed the study materials and conducted the analysis of the Spanish data. MV conducted the meta-analytic synthesis of studies. MF drafted the manuscript, and PG and MV provided critical revisions. All authors approved the final version of the manuscript for submission.

## Conflict of Interest Statement

The authors declare that the research was conducted in the absence of any commercial or financial relationships that could be construed as a potential conflict of interest.

## Footnotes

1 The original survey contained 15 neuromyths and 17 general knowledge questions about the brain. However, three of the neuromyths are actually true statements about the brain. In our analyses, we considered these three correct “myths” as part of the general knowledge questionnaire and restricted our use of the term “neuromyth” to the remaining 12 items (for a similar approach, see Gleichgerrcht et al., 2015). Furthermore, one of the original general knowledge questions (i.e., ‘learning is not due to the addition of new cells to the brain’) has been considered true in previous studies, although, as noted by Deligiannidi and Howard-Jones (2015) there is some scientific evidence against it (Leuner et al., 2002). In the present study we ignored this item. Consequently our questionnaire included 12 neuromyths and 19 general knowledge questions about the brain.
