# Supplementary material for: Neuromyths in Education: Prevalence among Spanish Teachers and an Exploration of Cross-Cultural Variation
Source: Front Hum Neurosci. 2016 Oct 13;10:496. doi: 10.3389/fnhum.2016.00496 (PMC5061738; doi:10.3389/fnhum.2016.00496)
Supplement: Supplementary file 1 [file Table_1.pdf]

Table S1. Characteristics of previous (and present) studies

Country / Source	Sample size	Sampling method
**United Kingdom** Dekker et al. (2012)	137	"The total sample of 242 participants included 137 teachers from the Dorset region of the UK and 105 teachers from several regions in the NL surrounding the Amsterdam area. Participants were primary school teachers (44%), secondary school teachers (50%), and other teachers (e.g., trainee teachers, teachers in special education, teaching assistants; 6%). The schools from which the teachers were drawn could be considered a random selection of primary and secondary schools in the UK and the NL." (p. 3)
**Netherlands** Dekker et al. (2012)	105	
**Greece** Deligiannidi & Howard-Jones (2015)	217	"Participants were 217 teachers employed in Athens and the Peloponnese region of Greece (155 females and 62 males) included 102 primary school teachers (47%) and 109 secondary school teachers (50%) and 6 teachers who worked in both types of school." (p. 3910)
**Turkey** Karakus et al. (2015)	278	"The research participants were primary and secondary school teachers. In this mixed-methods study, a total of, 278 teachers (124 primary and 154 secondary school teachers), whose ages ranged between 23 and 64 (M=36), participated to the first part of the study. 51.8% of participants were female and 48.2% was male. For the second part of the study, 3 female and 3 male (3 primary and 3 secondary school teachers) participants were randomly selected for in depth interviews." (p. 1934)
**Peru** Gleichgerrcht et al. (2015)	2222	"Participants were teachers of all levels currently working in Latin America. The total sample included 3,451 teachers from Argentina (n=551), Chile (n=598), Peru (n=2,222), and other Latin American countries (n=80, including Mexico, Nicaragua, Colombia, and Uruguay)." (p. 171)
**Argentina** Gleichgerrcht et al. (2015)	551	
**Chile** Gleichgerrcht et al. (2015)	598	
**Other Latin America** Gleichgerrcht et al. (2015)	80	
**China** Pei et al. (2015)	238	"Participants were 238 primary, secondary and high school teachers recruited in Shanghai, Shandong, Jiangsu and Zhejiang provinces." (p. 3682)
**Spain** Present study	284	See 'Method' section in the main text.
