# Supplementary material for: Neuromyths in Education: Prevalence among Spanish Teachers and an Exploration of Cross-Cultural Variation
Source: Front Hum Neurosci. 2016 Oct 13;10:496. doi: 10.3389/fnhum.2016.00496 (PMC5061738; doi:10.3389/fnhum.2016.00496)
Supplement: Supplementary file 2 [file Table_2.pdf]

Table S2. Prevalence (%) of neuromyths in previous (and present) studies

Item	UK	Netherlands	Greece	Turkey	Peru	Argentina	Chile	Other Latin America	China	Spain
Environments that are rich in stimulus improve the brains of pre-school children.	95	56	97	86.7	91.4	87.8	98.5	97.5	89	94
Individuals learn better when they receive information in their preferred learning style (e.g., auditory, visual, kinesthetic).	93	96	97	97.1	90.6	85.8	95.2	86.2	97	91.2
Exercises that rehearse co-ordination of motor-perception skills can improve literacy skills.	78	63	72	56.8	88.3	77.5	86.8	75	79	82
Short bouts of co-ordination exercises can improve integration of left and right hemispheric brain function.	88	82	56	72.3	77.8	73	81.3	87.5	84	77.1
Differences in hemispheric dominance (left brain, right brain) can help explain individual differences amongst learners.	91	86	71	78.8	74.7	57.9	81.3	73.3	71	67.3
It has been scientifically proven that fatty acid supplements (omega-3 and omega-6) have a positive effect on academic achievement.	69	54	50	79.1	76	58.3	66.6	58.8	14	45.1
We only use 10% of our brain.	48	46	45	50.4	67.5	56.1	41.5	60	59	44
Children are less attentive after consuming sugary drinks, and/or snacks.	57	55	48	43.9	56.3	31	51.5	55	62	33.8
There are critical periods in childhood after which certain things can no longer be learned.	33	52	24	67.3	67	71	74.2	66.2	14	29.9

Children must acquire their native language before a second language is learned. If they do not do so neither language will be fully acquired.	7	36	-	58.3	50	15.6	19.7	31.4	-	10.9
If pupils do not drink sufficient amounts of water (6–8 glasses a day) their brains shrink.	29	16	12	24.8	11.2	5.6	6	15	5	7.7
Learning problems associated with developmental differences in brain function cannot be remediated by education.	16	19	29	21.6	27.6	18.5	9.5	10	50	7
